# Análisis de las interacciones entre los actores de la Red de Gestión del Conocimiento, Investigación e Innovación en Malaria de Colombia

**DOI:** 10.7705/biomedica.6596

**Published:** 2022-12-01

**Authors:** Mayra Jiménez-Manjarrés, Diana M. Santana, Mario J. Olivera, Luz Stella Cantor- Poveda, Carlos A. Castañeda-Orjuela

**Affiliations:** 1 Observatorio Nacional de Salud, Instituto Nacional de Salud, Bogotá, D.C., Colombia Instituto Nacional de Salud Bogotá D.C Colombia; 2 Grupo de Parasitología, Instituto Nacional de Salud, Bogotá, D.C., Colombia Instituto Nacional de Salud Bogotá D.C Colombia

**Keywords:** malaria, epidemiología, gestión del conocimiento, análisis de redes sociales, procesamiento de texto, intercambio de información en salud, malaria epidemiology, knowledge management, social network analysis, word processing, health information exchange

## Abstract

**Introducción.:**

La malaria, o paludismo, es una enfermedad de gran impacto en la población colombiana, que debe ser abordada desde el punto de vista del trabajo en equipo de instituciones para el intercambio de conocimiento.

**Objetivo.:**

Analizar las interacciones de la Red de Gestión del Conocimiento, Investigación e Innovación en Malaria de Colombia.

**Materiales y métodos.:**

Se hizo un análisis de redes sociales que permitió identificar la proximidad entre los actores y el grado de conocimiento entre ellos; se observaron indicadores de densidad, diámetro, distancia media y centralidad de grado. El corpus documental para el estudio estuvo constituido por 193 documentos técnicos publicados entre el 2016 y el 2021, que fueron analizados empleando técnicas de procesamiento de texto mediante el lenguaje de programación R. La categorización de la red se realizó a partir de cinco variables: atención integral a pacientes, diagnóstico, epidemiología y sistemas de análisis de información en salud, política pública, y promoción y prevención.

**Resultados.:**

El análisis de las interacciones indicó que la red la conformaban 99 actores, de los cuales 97 (98 %), mostraron más interés en la producción de conocimientos en epidemiología y sistemas de análisis de información en salud, seguido de la categoría de atención integral a pacientes con 79 (80 %). El 54 % de los actores llevó a cabo estudios de promoción y prevención, siendo esta la categoría de menor abordaje.

**Conclusiones.:**

Este estudio contribuye al fortalecimiento de estrategias clave en la divulgación del conocimiento sobre la malaria en Colombia.

## Abstract (en)

**Introduction:**

Malaria is a disease with a high impact on Colombian population, which must be approached from the point of view of teamwork of institutions for knowledge exchange.

**Objective:**

To analyze the interactions of the *Red de Gestión del Conocimiento, Investigación e Innovación en Malaria de Colombia*.

**Materials and methods:**

An analysis of social networks was applied that allowed identifying the proximity between actors and the degree of knowledge between them. Indicators of density, diameter, average distance, and degree of centrality were observed. The documentary corpus for the study consisted of 193 technical documents published between 2016 and 2021, which were analyzed using text mining using the R programming language. The network was categorized based on five variables: comprehensive patient care, diagnosis, epidemiology and health information analysis systems, public policy and promotion and prevention.

**Results:**

The analysis of interactions indicated that the network was made up by 99 actors. The main interest in knowledge production was on epidemiology and health information analysis systems (98 % of the actors), followed by the integral patient care (80 % of the actors). On the contrary, the least approached category was malaria promotion and prevention practices (54 % of the actors).

**Conclusions:**

In general, this study contributes to the strengthening of key strategies in the dissemination of knowledge about malaria in Colombia.

La Organización Mundial de la Salud (OMS) considera que las dos últimas décadas han sido de éxitos sin precedentes en la lucha contra la malaria, con 1.5 millones de casos evitados y 7,6 millones de vidas salvadas en todo el mundo. Los países que eliminaron la malaria en su territorio han contribuido sustancialmente en la reducción de la carga mundial de la enfermedad.

La incidencia de casos de malaria se redujo de 81 por 1.000 habitantes en riesgo en el 2000 a 57 por 1.000 en el 2019 antes de aumentar nuevamente a 59 por 1.000 en el 2020. Se estima que el aumento en el año 2020 estuvo asociado con la interrupción de uno o más servicios en la atención de la malaria durante la pandemia de COVID-19. El 96 % de los casos de paludismo en todo el mundo corresponden a 29 países africanos. En las Américas, el 77 % de la carga total de malaria se registró en Brasil, Colombia y la República Bolivariana de Venezuela ^1^.

En Colombia, se registran 70.000 casos anuales, aproximadamente, con predominio de la infección por *Plasmodium vivax.* Se estima que cerca de 10 millones de personas se encuentran en riesgo de enfermar o morir por esta causa; principalmente en aquellas poblaciones que habitan en las regiones del Pacífico, la Amazonía y la Orinoquía, y los departamentos de Antioquia, Córdoba, Bolívar y Magdalena, y también, en la frontera con Venezuela ^2^^,^^3^.

El Plan Estratégico de Malaria en Colombia 2019-2022 se estableció con base en la Estrategia Técnica Mundial contra la Malaria 2016-2030, e incluye acciones para la prevención, el diagnóstico, el tratamiento y la mejora de la vigilancia epidemiológica de la malaria, para avanzar hacia su eliminación. Allí se indican las áreas en que las soluciones innovadoras serán esenciales para cumplir los objetivos y en las cuales el trabajo colaborativo en red será relevante ^4^^,^^5^.

En la última década, los procesos de globalización se han acelerado con importantes repercusiones para la ciencia y la tecnología ^6^. Por ello, los retos que plantea un mundo globalizado motivan a la sociedad a buscar nuevas soluciones para enfrentar de manera efectiva los desafíos emergentes, y replantear las formas de abordar los problemas endémicos de interés en salud pública, como la malaria ^7^.

En Colombia, se han promovido actividades científicas, tecnológicas y de innovación, que han incrementado el número de producciones científicas en malaria, y establecido la Red de Gestión de Conocimiento, Investigación e Innovación (Red Malaria), que tiene como objetivo facilitar el flujo y el intercambio de información, así como producir nuevos conocimientos y asegurar su aplicación implementando estrategias para involucrar a los tomadores de decisiones ^8^^,^^9^.

En el 2016, se conformó la Red Especializada del Conocimiento en Malaria como una iniciativa del Ministerio de Salud y Protección Social, el Instituto Nacional de Salud y la Organización Panamericana de la Salud, con la participación de entidades territoriales departamentales, distritales y municipales, instituciones prestadoras de servicios de salud, centros de investigación, la academia y la sociedad civil ^10^. Esta red ha gestionado el conocimiento en malaria, desarrollando estrategias para su creación y utilización, visualizando este conocimiento como un recurso estratégico para apoyar el cumplimiento de las metas de eliminación que se ha propuesto el país ^11^.

Se trata de determinar la productividad de la investigación sobre malaria y la manera como los diferentes actores e instituciones públicas y privadas se relacionan e interactúan al momento de generar producción científica, y considerar la toma de decisiones como parte de la gestión del conocimiento, ante la necesidad de crear líneas de investigación poco estudiadas y evaluar la interacción científica de autores e instituciones del país ^12^^,^^13^.

Las relaciones entre actores o autores de la producción científica se analizan mediante las menciones que realizan unos de otros. La teoría de redes sociales permite calcular la aproximación, la lejanía y la intermediación, así como la prevalencia, que son relevantes al momento de fortalecer el conocimiento de una enfermedad con tal grado de afectación en la población como la malaria ^14^^,^^15^.

Se han publicado pocos estudios bibliométricos sobre la investigación de la malaria en diferentes partes del mundo. Los primeros datan de los años 1984, 1989 y 1994, y posteriormente, entre los años 1996 y 2000, se estudió la financiación para la investigación sobre la malaria ^13^^,^^14^. Más recientemente, entre el 1972 y el 2003, se han producido investigaciones rigurosas en Brasil e India, en relación con la resistencia a los antipalúdicos, durante los años 2006 y 2015 ^13^^,^^15^. Sin embargo, ninguno de estos se ha llevado a cabo en Colombia. Por lo tanto, el objetivo del presente estudio fue analizar las interacciones de la Red Malaria en el último lustro, para contribuir en su fortalecimiento, identificando los actores que la conforman y los temas relevantes que se trabajan para la gestión del conocimiento en el tema de la malaria en Colombia.

## Materiales y métodos

En esta investigación se llevó a cabo un estudio cualitativo para categorizar el corpus documental identificado sobre malaria, y para la selección de un glosario de términos denominado “tesauro” ^16^, así como, la selección y organización de de unos documentos base de frecuente referencia llamados “acervos” ^17^. Además, se hizo un estudio de tipo cuantitativo para clasificar la producción bibliográfica recopilada, mediante el uso de algoritmos *k-means*, para el análisis de la medición de las diferentes categorías consideradas. El carácter del estudio se considera descriptivo con metodología bibliométrica ^18^.

La metodología del análisis consistió en la consolidación del listado de actores o autores relacionados con temas de malaria en Colombia, la identificación de categorías de estudio en malaria, la selección del glosario “tesauro” y de los “acervos”’ la búsqueda y clasificación del corpus documental por actor, y la identificación de las relaciones y el cálculo de medidas estadísticas empleando el análisis de redes sociales.

La consolidación del listado de actores o autores en Colombia relacionados con temas de malaria incluyó a los miembros de la Red Malaria, y a los autores encontrados en las bases de datos bibliográficas, incluyendo grupos de investigación, publicaciones, etc. Para la selección de las categorías de estudio en malaria, se tuvieron en cuenta los documentos base sobre el manejo y el estudio de la malaria, tales como: el Plan Estratégico Nacional de Malaria 2019-2022 ^4^; la evaluación del retorno de la inversión de la investigación en malaria financiada por Colciencias durante el periodo 1995-2005 ^19^; las directrices políticas de investigación, innovación científica y tecnológica y la gestión del conocimiento que contribuye a la eliminación de la malaria (2018) ^7^; la Iniciativa para la Eliminación de la malaria (IREM) en Mesoamérica y República Dominicana IREM ^20^; y la categorización de los énfasis de los proyectos de investigación en malaria financiados por Colciencias durante 1995-2005 ^21^.

También, se llevó a cabo una revisión exploratoria de la literatura científica, con el *software* Nvivo 12, con el fin de identificar *a priori* temas sobre la investigación de malaria en Colombia que pudieran ser el punto de partida para la generación de las categorías y, sobre todo, se tuvo en cuenta la opinión de expertos en el manejo, el análisis y el control de la malaria.

Se identificaron y utilizaron las categorías de estudio presentadas en el cuadro 1, para el análisis del corpus documental.

**Cuadro 1:** Categorías de análisis de la Red de Gestión de Conocimiento, Investigación e Innovación en Malaria en Colombia 2021

Nombre de categoría	Temas incluidos en la categoría
Atención integral al paciente	Atención de pacientes, guías en el manejo del paciente hospitalario e intrahospitalario, respuesta, redes de prestación de servicios de salud, características de la atención de salud, medidas para la atención en presencia de malaria, diagnóstico muy general de la malaria
Diagnóstico	Diagnóstico, suministros, pruebas, síntomas, detección, clínica, tratamiento, fisiopatología de la malaria, resistencia a los medicamentos antimaláricos, genética del parásito, vacunas contra la malaria
Epidemiología y sistema de información de análisis en salud	Epidemiología y sistema de información de análisis en salud
Política pública	Política pública, economía y aspectos sociales de la malaria
Promoción y prevención	Promoción, prevención, entomología y control de vectores

Una vez identificadas las categorías como temas de interés para la red de conocimiento en malaria, se procedió a seleccionar los términos característicos (tesauros) y los documentos distintivos (acervos) de cada una de ellas. Los términos y documentos permiten cotejar y clasificar el corpus documental en las categorías mencionadas en el cuadro 1, mediante la aplicación de algoritmos *k-means* que, a su vez, van estructurando una base de datos con las relaciones de entrada y salida de nodos; estos últimos representan los autores o actores de la red de conocimiento.

La población de estudio incluyó la producción científica relacionada con malaria, en idioma español y en formato pdf, que las instituciones públicas y privadas de Colombia generaron entre enero de 2016 y marzo de 2021. Se incluyeron artículos de investigación o revisión, documentos técnicos, documentos de política, capítulo de libro o libro completo, boletín técnico, estudios de caso, tesis y trabajos académicos, monografías, revisión de productos o memorias de seminarios.

Se excluyeron del estudio los siguientes: cartas al editor, resúmenes de presentaciones en congresos, infografías y folletos, documentos normativos y textos legales.

La técnica de recolección de los documentos fue la búsqueda por actor en bases de datos bibliográficas como Scopus y Google Scholar, así como en los repositorios institucionales y mediante solicitudes por correo electrónico a los actores que hacen parte de la red de malaria.

### Procesamiento y análisis

Una vez identificadas las categorías, seleccionados los tesauros y los acervos, y recopilado el corpus documental, se procedió al almacenamiento y carga de toda la información en el código creado, para facilitar la clasificación de los documentos en categorías y la identificación de las relaciones entre actores. Este proceso se llevó a cabo en el lenguaje R con *Rstudio* como entorno de desarrollo integrado.

Las salidas de la carga del corpus documental en el código dieron como resultado una tabla que contiene las relaciones entre actores en cada una de las categorías identificadas en la producción bibliográfica. A partir de esta tabla, se calcularon las medidas estadísticas utilizadas para el análisis cuantitativo de la red de conocimiento en malaria. Estas medidas correspondieron a:

- Número de nodos: indica el número de actores o instituciones que intervinieron en la red de conocimiento ^22^.
- Número de enlaces: indica el número de conexiones o aristas que relacionan los nodos ^23^.
- Centralidad de grado: indica si una red es popular, independiente y con diversas formas de satisfacer las necesidades de los actores ^24^.
- Densidad: propiedad que calcula la proporción de los enlaces presentes en la red sobre el máximo número de enlaces que pueden existir; la red es poco densa cuando no existen relaciones entre los actores, y es muy densa cuando todos los actores están relacionados entre sí ^22^.
- Diámetro: menor número de enlaces recorridos entre los dos actores más alejados de la red ^25^.
- Distancia o longitud media del camino: media de las distancias entre todos los pares posibles de nodos; corresponde a una medida de la eficiencia de la información o el transporte masivo en una red ^26^.
- Por nodo o actor, se calcularon las siguientes medidas estadísticas métricas:
- Grado de entrada de un nodo: número de enlaces que apuntan hacia él y centralidad del vector propio; mide la influencia de un actor en una red ^27^.
- Excentricidad: medida que brinda la distancia entre un nodo y el nodo que está más alejado de él ^25^.
- Cercanía (*closseness*): mide la accesibilidad de un nodo ^24^.
- Intermediación (*betweenness*): cuantifica la frecuencia o el número de veces que un nodo actúa como un puente a lo largo del camino más corto entre otros dos nodos ^15^.

Para facilitar el análisis, se desarrolló en Power BI pro una visualización que permite apreciar las características de la red, así como las medidas por categoría y por nodo o actor, en cada categoría. La red se visualiza en el siguiente enlace: https://app.powerbi.com/view?r=eyJrIjoiYjMwMzYyZDItNjkwOC00MTQ1LWIxNWYtZTRhZjUxNm ExOD c3IiwidCI6ImE2MmQ2YzdiLTlmNTktNDQ2OS05MzU5LTM1M zcxNDc1OTRiYiIsImMiOjR9

## Resultados

### *Análisis entre las categorías de la red de malaria*

La red se constituyó a partir de 193 documentos de producción bibliográfica sobre malaria y se identificaron relaciones entre 99 nodos o autores.

En el cuadro 2, se observa que las categorías con mayor número de enlaces, nodos o actores y centralidad de grado son: epidemiología y sistema de análisis de información de salud y atención integral al paciente, respectivamente, lo cual significa que son los temas sobre malaria más abordados en Colombia. Estas dos categorías, junto con la política pública, tienen menor distancia media, lo cual significa que hay una mayor eficiencia en la difusión de la información dentro de cada categoría con respecto a prevención, promoción y diagnóstico, que tienen mayor valor en esta medición. La categoría de política pública presentó una densidad superior porque, a pesar de identificarse la participación de 79 nodos, estos se relacionan con 317 enlaces, lo cual significa que están conectados.

**Cuadro 2:** Métricas por categoría de la Red de Malaria

Categoría	Densidad	Diámetro	Nodos	Enlaces	Distancia media	Centralidad de grado
Promoción y prevención	0,04	3	53	114	2,56	0,19
Diagnóstico	0,04	6	70	184	2,57	0,20
Atención integral al paciente	0,04	4	88	319	2,36	0,26
Epidemiología y sistema de información de análisis en salud	0,04	6	97	397	2,31	0,26
Política pública	0,05	4	79	317	2,19	0,30

### Análisis por categoría de la Red Malaria

**Figura 1:**
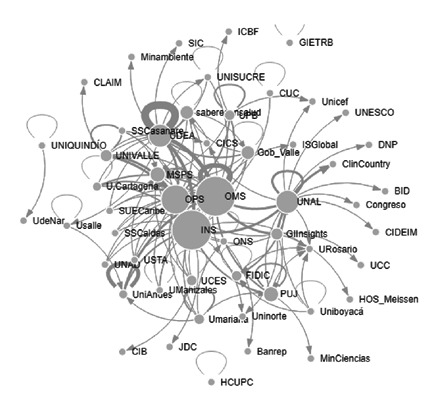
Interacción de actores en la categoría de promoción y prevención en malaria

Para la categoría de “promoción y prevención de malaria”’ los nodos o actores con mayor número de menciones fueron: la Organización Mundial de la Salud (OMS) ^23^, el Instituto Nacional de Salud ^19^, la Organización Panamericana de la Salud (OPS) ^15^, el Ministerio de Salud y Protección Social ^8^, y la Universidad de Antioquia ^6^. Los nodos cercanos a otros, detectados en esta categoría de la red, fueron: el Instituto Nacional de Salud (0,16), la OMS (0,16), el Ministerio de Salud y Protección Social (0,16), la Gobernación del Valle (0,16), la Corporación Saberes en Salud (@saberesensalud) (0,16) y la OPS (0,16). Los nodos con mayores valores en centralidad del vector propio fueron: la OMS (1,00), la Universidad de Antioquia (0,89), el Instituto Nacional de Salud (0,86), la OPS (0,64), la Universidad Nacional (0,48); y la Universidad del Valle (0,40), por lo cual en la figura 1 se aprecian como los nodos más grandes. Los nodos intermediarios de la categoría fueron: el Instituto Nacional de Salud (0,03), la Universidad de Antioquia (0,01), la Universidad Nacional (0,01), la OmS (0,01) y la Pontificia Universidad Javeriana (0,01). Según los indicadores, los nodos que sobresalen como intermediario, también sobresalen como influenciadores.

**Figura 2:**
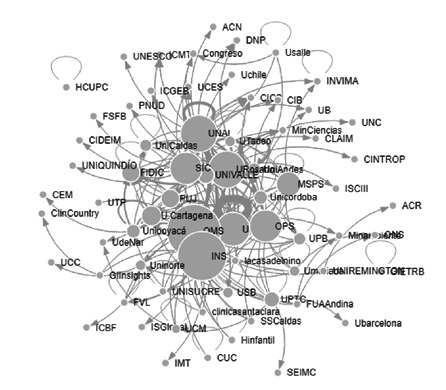
Interacción de actores en la categoría de diagnóstico de malaria

Para la categoría de “diagnóstico de malaria’ los nodos con mayor número de menciones fueron: la OMS ^31^, el Instituto Nacional de Salud ^23^, la OPS ^17^, la Universidad de Antioquia ^11^, el Ministerio de Salud y Protección Social (10), y la Fundación Instituto de Inmunología de Colombia ^8^. Los nodos con mayor valor en cercanía detectados en esta categoría de la red, fueron: la OMS (0,26), el Instituto Nacional de Salud (0,26), la Superintendencia de Industria y Comercio (0,25), la Universidad de Cartagena (0,25), el Ministerio de Salud y Protección Social (0,24), la Fundación Instituto de Inmunología de Colombia (0,24), la Pontificia Universidad Javeriana (0,24) y la OPS (0,24). Los nodos con mayores valores en centralidad del vector propio fueron: la OMS (1,00), la Universidad Nacional (0,93), la Universidad de Antioquia (0,92), el Instituto Nacional de Salud (0,84), la Universidad del Rosario (0,69), y la OPS (0,62). Con una centralidad del vector propio en 0,00 quedaron aproximadamente 11 actores, los cuales se aprecian en los extremos del gráfico de la figura 2 , entre los que aparecen la Asociación Colombiana de Radiología; la Clínica del Country, la Universidad Cooperativa y el Instituto Colombiano de Bienestar Familiar, entre otros. Los nodos intermediarios de la categoría “diagnóstico en malaria” son: el Instituto Nacional de Salud (0,09) la Universidad de Antioquia (0,06), la Universidad del Rosario (0,06), la Universidad Nacional (0,06), el Ministerio de Salud y Protección Social (0,06), la OMS (0,03), la OPS (0,03), la Pontificia Universidad Javeriana (0,02), la Universidad de Cartagena (0,01) y la Fundación Instituto de Inmunología de Colombia (0,01).

En la categoría “atención integral al paciente”, los nodos con mayor número de menciones en la producción bibliográfica fueron: la OMS ^33^, el Instituto Nacional de Salud ^26^, la OPS ^24^, el Ministerio de Salud y Protección Social ^18^ y la Universidad de Antioquia ^17^. La OMS ^2^, con menor excentricidad, puede ser considerado el actor central de la categoría.

**Figura 3:**
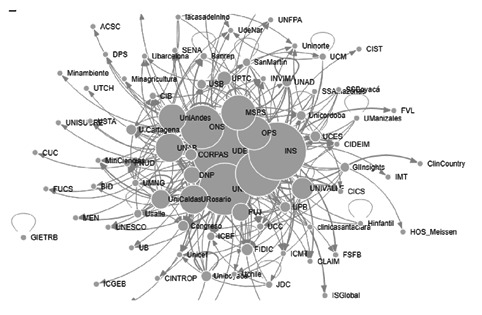
Interacción de actores en la categoría de atención integral al paciente

Los nodos detectados como cercanos en la figura 3, de “atención integral al paciente” fueron: la Universidad Nacional (0,33), la Universidad de Antioquia (0,32), la Universidad Autónoma de Bucaramanga (0,32), la CORPAS (0,31), la Universidad Pedagógica y Tecnológica de Colombia (0,31) y la Universidad de Caldas (0,31). Los nodos con mayores valores en centralidad del vector propio fueron: el Instituto Nacional de Salud (1,00), la Universidad Nacional (0,93), la OMS (0,87), la Universidad de Antioquia (0,80), la OPS (0,70) y el Ministerio de Salud y Protección Social (0,66). Los nodos intermediarios de la categoría fueron: la Universidad de Antioquia (0,11), el Instituto Nacional de Salud (0,10), la Universidad Nacional (0,08), la Universidad de los Andes (0,05) y el Observatorio Nacional de Salud (0,05). La mayoría del resto de los actores presenta un indicador en cero en esta medición.

**Figura 4:**
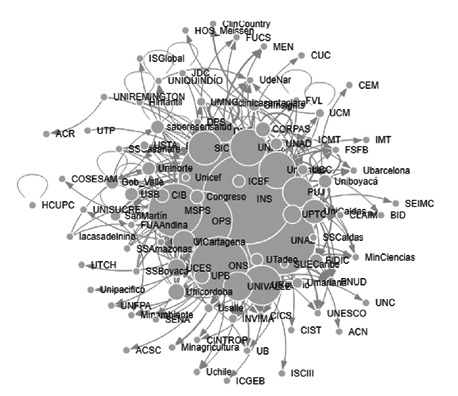
Interacción de actores en la categoría de epidemiología y sistemas de información de análisis de salud en malaria

En la categoría “epidemiología y sistemas de información de análisis de salud”’ los nodos con mayor número de menciones fueron: la OMS (44), el Instituto Nacional de Salud (39), la OPS (34), el Ministerio de Salud y Protección Social ^24^, y la Universidad de Antioquia ^21^. El actor central en esta categoría sería la OMS ^2^. Los nodos con mayor valor en cercanía detectados en la categoría “atención integral al paciente” fueron: la Universidad de Antioquia (0,33), la Superintendencia de Industria y Comercio (0,33); el Instituto Nacional de Salud (0,32), la Fundación Universitaria Juan N. Corpas (0,32, la Universidad Autónoma de Bucaramanga (0,32); la Universidad Nacional (0,31) y la Universidad Pedagógica y Tecnológica de Colombia (0,31). Con menor valor en cercanía para esta categoría, se ubicó el Hospital César Uribe Piedrahita de Caucasia, hecho que se aprecia de manera aislada en la figura 4.

Los nodos con mayores valores en centralidad del vector propio fueron: la Universidad Nacional (1,00), el Instituto Nacional de Salud (0,93), la OMS (0,89), la Universidad de Antioquia (0,82) y la OPS (0,69). Los nodos intermediarios respecto a la “atención integral al paciente” fueron: el Instituto Nacional de Salud (0,16), la Universidad de Antioquia (0,14), la Universidad Nacional (0,09), la Universidad de los Andes (0,07) y el Observatorio Nacional de Salud (0,06).

**Figura 5:**
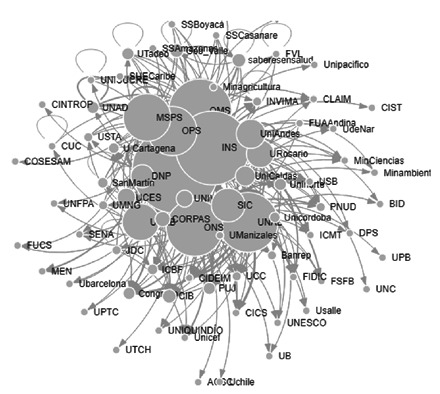
Interacción de actores en la categoría de política pública

Sobre la categoría “política pública”’ los nodos con mayor número de menciones fueron: la OMS (36), el Instituto Nacional de Salud ^28^, la OPS ^28^, el MSPS ^20^, y la Universidad de Antioquia ^18^, a los cuales ingresan un mayor número de enlaces, como se muestra en la figura 5.

De igual manera, con menor valor en excentricidad se registraron: el Instituto Nacional de Salud ^2^ el Ministerio de Salud y Protección Social ^2^, la OMS ^2^, la OPS ^2^ y la Universidad de Antioquia ^2^. Los nodos más cercanos detectados en la categoría de la red, fueron: la Universidad de Antioquia (0,55), la Superintendencia de Industria y Comercio (0,51), la Universidad Nacional (0,51), la Universidad de los Andes (0,50) y el Instituto Nacional de Salud (0,49). Los nodos con valores mayores en centralidad del vector propio fueron: la Universidad Nacional (1,00), el Instituto Nacional de Salud (0,92), la OMS (0,82), la Universidad de Antioquia (0,78) y la OPS (0,72). Respectivamente, los nodos intermediarios fueron: la Universidad de Antioquia (0,17), el Instituto Nacional de Salud (0,12), el Observatorio Nacional de Salud (0,06) y la Universidad Nacional (0,06). Por obtener los mayores valores, sin embargo, la mayoría de los nodos no fueron intermediarios con valores en cero y cercanos.

Analizando los actores no mencionados, en todas las categorías se encontraron, al menos, siete nodos que generaron contenido bibliográfico sobre malaria, pero que no fueron referenciados. Las categorías de “promoción y prevención’,’ y, “política pública” tuvieron menor número de nodos con alto grado de excentricidad, lo cual significa que tienen menor número de nodos periféricos.

Con menor excentricidad, sobresalieron los mismos actores con mayor número de menciones, coincide con que serían los actores centrales de cada una de las categorías, sobresaliendo la OMS como actor central (con menor valor de excentricidad) en tres categorías: “atención integral al paciente” “diagnóstico y epidemiología” y “sistema de información de análisis de salud”

En cuanto a nodos cercanos, cada categoría tuvo su distinción. No obstante, el actor que estuvo cercano en todas las categorías, excepto en “atención al paciente en malaria” fue el Instituto Nacional de Salud. Se evidenció que las universidades se caracterizaron por ser actores cercanos en todas las categorías, excepto en la de “promoción y prevención” Como actores influyentes en todas las categorías, se incluyen los actores centrales o los más nombrados, así como también, centros educativos como la Universidad Nacional, la Universidad de Antioquia, la Universidad del Rosario y la Universidad del Valle.

## Discusión

En el presente estudio, la aplicación de la bibliometría permitió identificar las interacciones y los actores clave en la producción científica de la Red Malaria en Colombia. Los resultados de este análisis de redes sociales contribuirán al diseño de estrategias que incentiven el fortalecimiento y expansión de esta red de conocimiento especializada en malaria.

Un hallazgo relevante en este estudio fue que el tema de la prevención y promoción de la malaria presentó menor número de conexiones y actores. Esto significa que es una temática que debe ser abordada más eficientemente, de modo que los actores puedan mejorar en el proceso de divulgación del conocimiento sobre las medidas de eliminación y prevención de esta enfermedad.

Este resultado podría explicarse por la dificultad para trabajar de manera articulada entre la academia, el Estado y la comunidad. Los proyectos de investigación que desarrollan esta línea de trabajo, generalmente son iniciativas de instituciones gubernamentales que incluyen la participación comunitaria para el abordaje integral de esta problemática en salud y que afectan en los municipios, temas como el saneamiento básico, el acceso al servicio de salud, la falta de educación y la fragilidad de infraestructura, entre otros ^28^.

Otro hallazgo relevante se presentó en todas las categorías temáticas con parámetros de densidad similares. Desde la teoría de redes se puede inferir que se encuentran en condiciones similares de conectividad entre sus actores y que todos ellos, en todas las categorías, podrían aumentar su capacidad de comunicación o cooperación entre ellos, por medio de estrategias como: talleres, seminarios, congresos, colaboración en artículos científicos, intercambio de ideas, o asesorías (Lopera Lopera, Luis Hernando Integración de redes de conocimiento: una responsabilidad de la biblioteca universitaria, 2000. Sexto Congreso Nacional de Bibliotecología y Documentación, ASCOLBI, Santafé de Bogotá (Colombia), 4-7 July, 2000).

Analizando las categorías desde una perspectiva global, los actores más citados y que coincidieron en todas las categorías, fueron: la OMS, el Instituto Nacional de Salud, la OPS, el Ministerio de Salud y Protección Social, y la Universidad de Antioquia. Estos actores tienen en común ser entidades nacionales e internacionales de referencia en la gestión del conocimiento en salud.

Por otro lado, centros educativos como la Universidad Nacional, la Universidad de Antioquia, la Universidad del Rosario y la Universidad del Valle, son las entidades que comúnmente participan de manera activa en actividades de investigación en salud pública, y son considerados actores centrales en la presente investigación.

Los hallazgos de estas interacciones deberían permitir impulsar el trabajo colaborativo de las instituciones, para que mejoren las relaciones en pro de la creación y aplicación del conocimiento para lograr la eliminación de la malaria.

Un análisis bibliométrico, publicado en el 2021, sobre la investigación de la malaria en Latinoamérica coincide con las categorías temáticas de estudio más y menos abordadas en Colombia: la epidemiología y la prevención ^29^. A pesar de que cada estudio tiene sus criterios de clasificación, también hay coincidencia en la escogencia de tres temas como categorías de análisis: epidemiología, diagnóstico y prevención de la malaria. En el mismo estudio, se menciona a Colombia como el segundo país con mayor número de publicaciones sobre malaria, y a la afiliación a la Universidad de Antioquia como la segunda más frecuente en Latinoamérica. Este resultado coincide con el presente estudio en el que la Universidad de Antioquia es uno de los actores más citados y activos en la producción de conocimiento sobre malaria en Colombia.

Entre las recomendaciones de este estudio se plantea tener en cuenta qué actores se clasifican como influenciadores (con alto valor en centralidad del vector propio) e intermediarios, ya que pueden ser clave en la difusión de eventos, información y divulgación del conocimiento. Especialmente, los intermediarios pueden generar conexiones entre grupos de actores para asociarse en investigaciones y en la realización de actividades de gestión del conocimiento.

También, se recomienda tener en cuenta aquellos autores que están incursionando en las temáticas en malaria, porque tienen poca o ninguna mención. Esto representa una oportunidad para que sean integrados en la red de conocimiento y puedan dar a conocer el potencial de aportes que tienen. Esto significa que estos actores deben dar a conocer su producción documental para que sean tenidos en cuenta en la apropiación y divulgación del conocimiento, dependiendo de la categoría de análisis.

Teniendo en cuenta otras metodologías de búsqueda del corpus documental para el análisis bibliométrico, se puede evidenciar que existe la posibilidad de encontrar mayor producción bibliográfica por medio de búsquedas sistemáticas bastante específicas, en bases de datos y en revistas científicas, como Scopus ^29^ o *Infectio*^30^*,* respectivamente*.* Sin embargo, el número de textos analizados también se relaciona con el período considerado para la recopilación de información y la posibilidad de analizar documentos en idiomas diferentes al español en dichas metodologías.

En el marco de los objetivos misionales del Instituto Nacional de Salud y del Ministerio de Salud y Protección Social, no solo ha estado la conformación de las redes de conocimiento en salud pública, incluida la Red de Gestión de Conocimiento, Investigación e Innovación en Malaria, sino también, su fortalecimiento incentivando a otras instituciones e investigadores a vincularse y aportar los productos esperados en cada red ^31^. El esfuerzo del Instituto Nacional de Salud se ve reflejado en estrategias diseñadas para favorecer una constante comunicación entre actores. Una de ellas es el mantenimiento de plataformas y medios digitales ^31^. Esto ha hecho posible un liderazgo que ha fortalecido la cooperación, fomentando una integración constructiva y creativa ^32^. Además, la pandemia por SARS-CoV-2 representó una valiosa oportunidad que permitió ampliar el uso de nuevas herramientas de difusión de la información y crear nuevos espacios para la interacción entre los actores ^33^.

Entre las limitaciones de esta investigación se puede mencionar el posible sesgo al momento de recopilar documentos sobre el tema de la malaria. Aunque la producción científica se solicitó a los actores por correo electrónico y se hizo una búsqueda activa en repositorios, páginas web y bases de datos, siempre existe la posibilidad de que algunos documentos queden por fuera de los análisis. En segundo lugar, la metodología limita la clasificación en categorías al basarse únicamente en documentos en español. Otra posible limitación está en la definición de las categorías de malaria, porque existe la posibilidad de definirlas y seleccionarlas por medio de múltiples estrategias de análisis cualitativo. Sin embargo, cabe destacar que los expertos en malaria intervinieron en la aprobación de los temas por analizar con la red, por lo cual se representa el interés de quienes son los líderes en la red de malaria.

## References

[1] World Health Organization (2021). World Malaria Report 2021.

[2] Instituto Nacional de Salud - Sivigila (2019). Informe de evento malaria.

[3] Padilla-Rodríguez JC, Olivera MJ, Ahumada-Franco ML, Paredes-Medina AE. (2021). Malaria risk stratification in Colombia 2010 to 2019. PLoS ONE.

[4] Ministerio de de Salud y Protección Social (2020). Plan Estratégico Nacional de Malaria 2019-2022.

[5] World Health Organization (2015). Global technical strategy for Malaria 2016-2030.

[6] United Nations Educational Scientific and Cultural Organization, UNESCO (2015). Science Report: Towards 2030.

[7] Instituto Nacional de Salud (2018). Lineamiento político de investigación, innovación científica y tecnológica y gestión del conocimiento que contribuye a la eliminación de la malaria 2018.

[8] Instituto Nacional de Salud (2018). Medidas de intervención para la constitución de la red de gestión del conocimiento, investigación e innovación en malaria.

[9] Ministerio de Salud y Proteccion Social (2015). Orientaciones para el desarrollo de la información en salud en el marco del Plan de Salud Pública de Intervenciones Colectivas - PIC.

[10] Martínez E, Franco D, Villa L. (2009). Las redes de conocimiento en salud pública y el fortalecimiento de capacidades a través de estrategias de cooperación. Rev Fac Nac Salud Pública.

[11] Ministerio de de Salud y Protección Social (2020). Guía para la gestión por procesos en el marco del modelo integrado de planeación y gestión.

[12] Otálora-Escorcia A. (2008). El análisis bibliométrico como herramienta para el seguimiento de publicaciones científicas, tesis y trabajos de grado.

[13] Mac Clean M, Anderson J, Davies C. (1997). Making malaria research bite. Nature.

[14] Lewison G, Lipworth S, Francisco A. (2002). Input indicators from output measures: A bibliometric approach to the estimation of malaria research funding. Research Evaluation.

[15] Sweileh WM, Al-Jabi SW, Sawalha AF, AbuTaha AS, Zyoud SH (2017). Bibliometric analysis of worldwide publications on antimalarial drug resistance (2006-2015). Malar Res Treat.

[16] Martínez-Méndez FJ, Pastor-Sánchez JA. (2003). Gestión colaborativa de tesauros en Internet.

[17] Archivo General de la Nación (2013). Acervo documental.

[18] Romani F, Huamani C, González G. (2011). Estudios bibliométricos como línea de investigación en las ciencias biomédicas: una aproximación para el pregrado. CIMEL: Ciencia e Investigación Médica Estudiantil Latinoamericana.

[19] Macías-Ángel B del P. (2015). Evaluación del retorno de la inversión de la investigación en malaria financiada por Colciencias durante el periodo 1995 - 2005.

[20] Ministerio de Salud Pública (2017). Iniciativa Regional para la Eliminación de la Malaria en Mesoamérica y República Dominicana - IREM.

[21] Macías-Angel B, Agudelo CA, Ronderos-Torres MM. (2017). Categorización de los énfasis de los proyectos de investigación en malaria financiados por Colciencias durante 1995-2005. Rev Salud Pública.

[22] Universidad ICESI (2019). Breve tutorial para visualizar y calcular métricas de Redes (grafos) en R (para Economistas).

[23] Vidal-Ledo M, Vialart M, Hernández G. (2013). Redes sociales. Educación Médica Superior.

[24] Prada-Madrid E. (2005). Las redes de conocimiento y las organizaciones. Revista Bibliotecas y Tecnologías de la Información.

[25] Humberstone J. (2019). Análisis de redes sociales: Identificación de comunidades virtuales en Twitter. Real y Reflexión.

[26] Ander J. (2019). Análisis e implementación de redes de interconexión de diámetro baj.

[27] Ruhnau B. (2000). Eigenvector-centrality - a node-centrality. Soc Networks.

[28] Knudson-Ospina A, Barreto-Zorza M, Castillo C, Mosquera L, Apráez-Ippolito G, Olaya- Másmela L (2019). Estrategias para la eliminación de la malaria: una perspectiva afro- colombiana. Rev Salud Pública.

[29] Briceño-Gómez C, Tapia-Sequeiros G, Torreblanca-Rodríguez SM, Valdivia-Vargas L, Aquino-Canchari CR. (2021). Scientific research on malaria: A bibliometric analysis in Latin America, 2011-2020. Boletín de Malariología y Salud Ambiental.

[30] Suárez JO. (2012). Análisis bibliométrico de la revista Infectio, 1995 a 2011. Infectio.

[31] Instituto Nacional de Salud Plataforma Web Redes del conocimiento.

[32] Instituto Nacional de Salud de Colombia Medidas de intervención para la constitución de la red de gestión de conocimiento, investigación e innovación en malaria.

[33] Agudelo M, Chomali E, Suinaga J, Núñez G, Jordán V, Rojas F (2020). Las oportunidades de la digitalización en América Latina frente al Covid-19. Corporación Andina de Fomento, Naciones Unidas.

